# Supplementary material for: Developmental mouse brain common coordinate framework
Source: Nat Commun. 2024 Oct 21;15:9072. doi: 10.1038/s41467-024-53254-w (PMC11494176; doi:10.1038/s41467-024-53254-w)
Supplement: Supplementary file 1 — Supplementary Information [file 41467_2024_53254_MOESM1_ESM.pdf]

Supplementary Information

Supplementary Figure 1. DevCCF templates enhanced by averaging individual samples

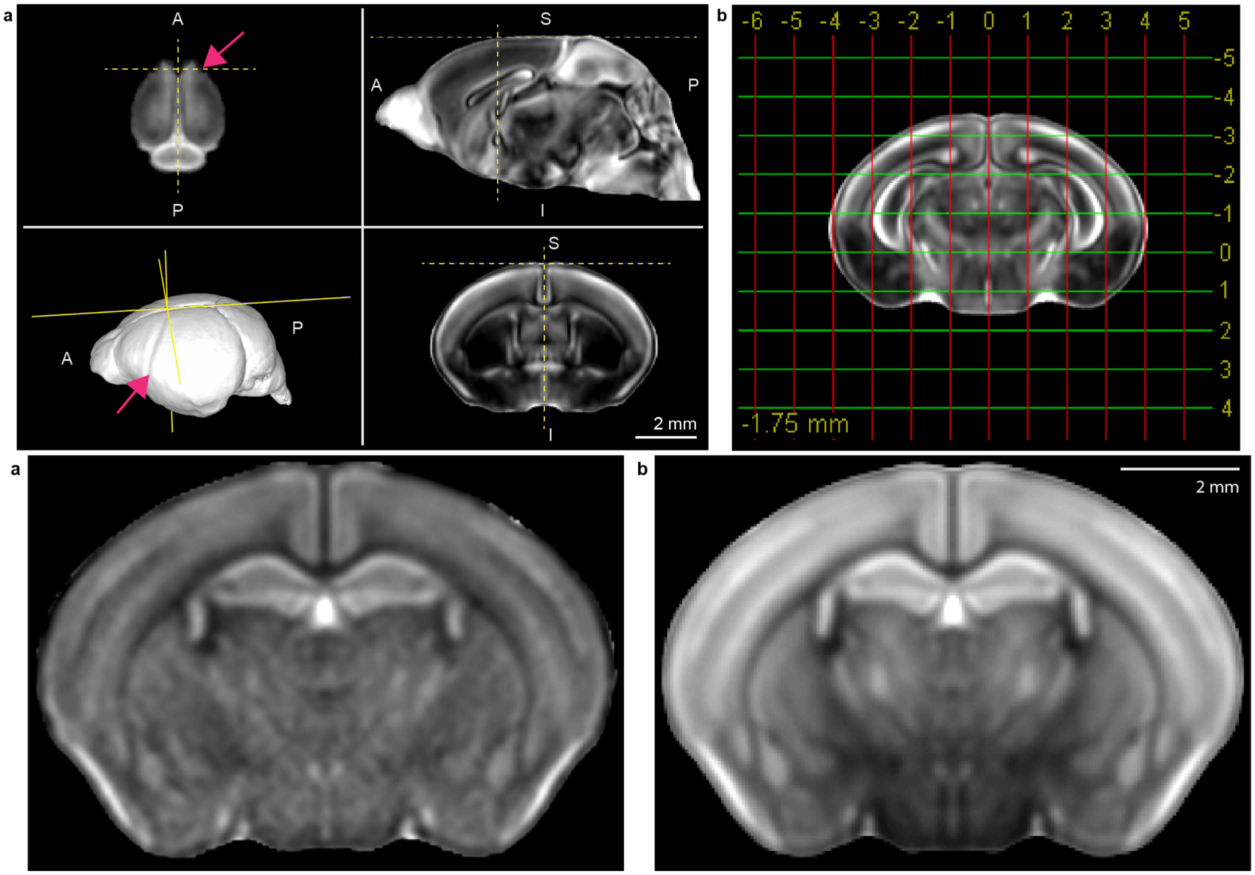

(a) Individual P14 MRI ADC sample warped to P14 DevCCF has distinguishable features including cortical layers. (b) P14 morphological average MRI ADC template displays refined convergence of anatomical features in (a), with additional features clearly visible, such as thalamic nuclei. Scale bar = 2 mm.

Supplementary Figure 2. Stereotaxic Coordinates in the templates

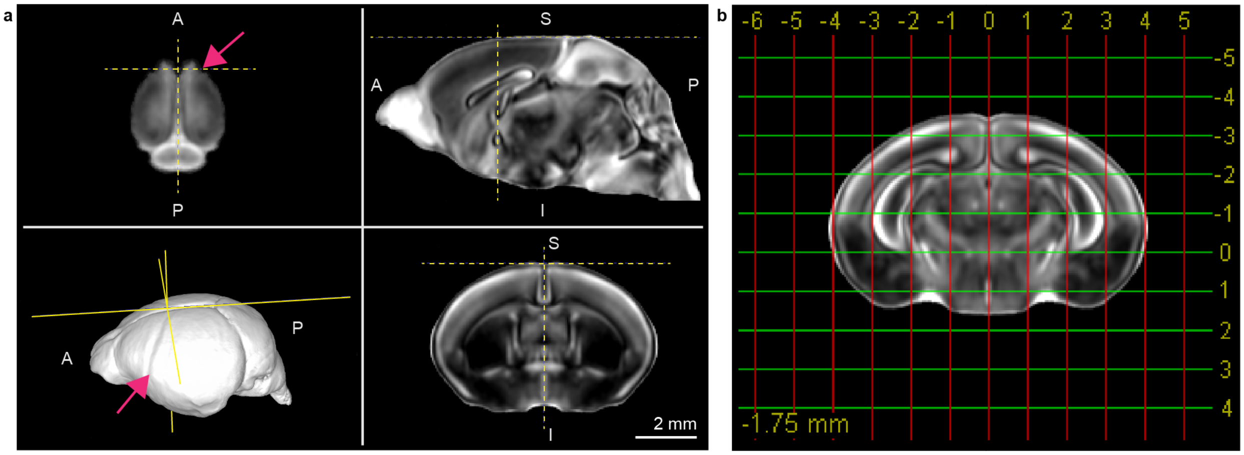

Postnatal atlases were aligned to stereotaxic coordinates with the anterior commissure and estimated bregma. (a) FA templates are visualized in 2D orthogonal planes, with the T2 template surface visualized as a 3D rendering. The FA template clearly shows the anterior commissure. The T2 template shows a cortical impression used to estimate the bregma (magenta arrow). Yellow lines represent the location of orthogonal planes. (b) Stereotaxic grid overlayed on P4 MRI FA template as labels. Each color represents a different label (red-sagittal spacing; green-horizontal spacing; yellow-spacing labels). A: anterior, P: posterior, S: superior, I: inferior.

Supplementary Figure 3. ADMBA pallium compared to concentric ring topology

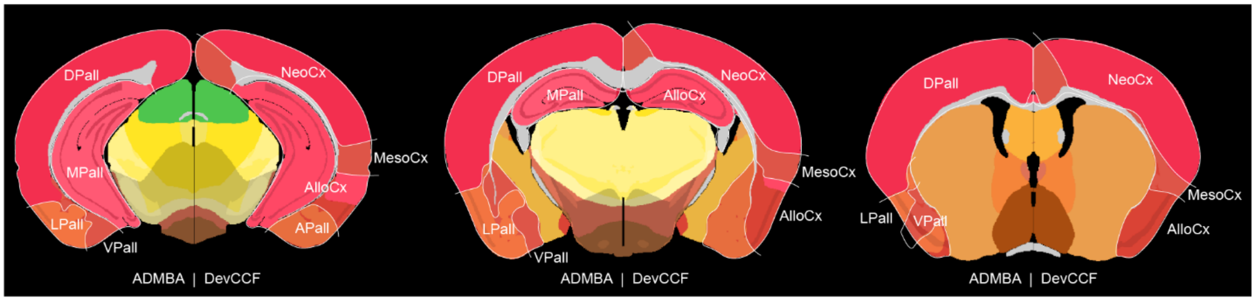

Coronal slices comparing the pallium as segmented by ADMBA ontology (left hemisphere) and DevCCF ontology (right hemisphere). Annotation colors defined in DevCCF ontology (Supplementary Data 2). White lines show pallial delineations. ADMBA ontology divides the pallium as dorsal (DPall), medial (MPall), lateral (LPall), and ventral (VPall). In contrast, concentric ring topology represented by the DevCCF divides the pallium into the neocortex (NeoCx), Mesocortex (MesoCx), allocortex (AlloCx), and Pallial Amygdala (APall).

Supplementary Figure 4. Validation of DevCCF annotations by gene expression

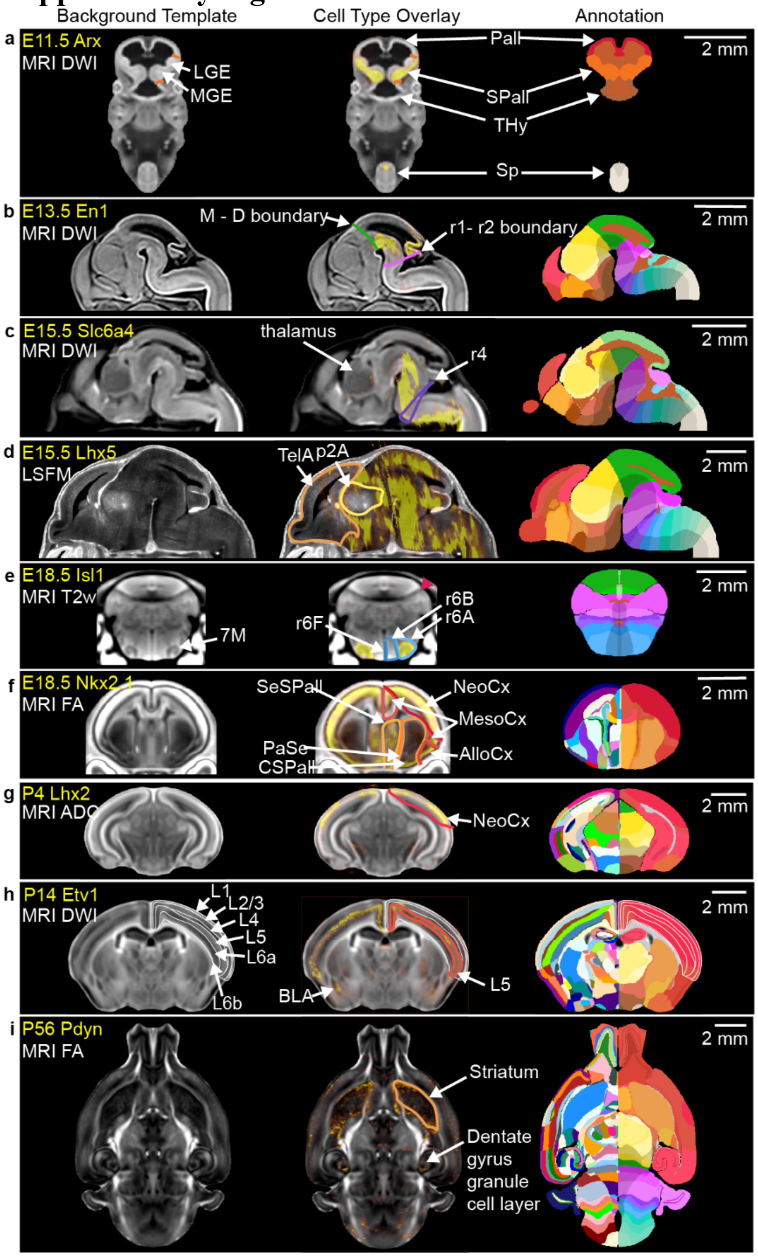

Each panel represents a DevCCF template, spatial gene expression profile (yellow) overlayed on the template (greyscale), and associated DevCCF annotations. Annotations split by hemisphere (f-i) represent DevCCF colors on right and semi-randomized colors on left for visualization. Delineation color over a template represents the assigned DevCCF ontology color. (a) E11.5 Arx validates SPall segmentation via boundaries with Pall and THy. (b) E13.5 En1 validates the M/D and r1/r2 boundaries. (c) E15.5 Slc6a4 validates r4 via its absence. (d) E15.5 Lhx5 similarly validates TelA and p2A. (e) E18.5 Isl1 is colocalized with 7M, validating gene expression alignment and the r6A (lightest blue outline) boundaries. The red arrowhead indicates the brain surface of the brain between M and Cb. (f) E18.5 Nkx2.1 expression highlights the superficial cortex layer in coherence with the background template. Additionally, Nkx2.1 highlights SeSPall. CSPall is delineated from SPall lateral to the ventricle. PaSe is next to the ventricle

between CSPall and SeSPall. (g) P4 Lhx2 L2/3 and L4 expression outlines NeoCx boundaries. (h) P14 Etv1 expression localizes L5 parcellation. White lines outline the cortical layers. (i) P56 MRI FA template highlights white matter tracts. Pdyn validates the striatum and dente gyrus granule cell layer segmentation. Scale bars = 2 mm. Abbreviations: allocortex (AlloCx), basolateral amygdala (BLA), cerebellum (Cb), central subpallium (CSPall), cortical layer (L), diencephalon (D), facial motor nucleus (7M), lateral geniculate nucleus (LGE), medial geniculate nucleus (MGE), mesocortex (MesoCx), midbrain (M), neocortex (NeoCx), pallium (Pall), paraseptal subpallium (PaSe), rhombomere # (r#), r6 alar plate (r6A), r6 basal plate (r6B), r6 floorplate (r6F), subpallium (SPall), septal SPall (SeSPall), spinal cord (Sp), telencephalon (TelA), terminal hypothalamus (THy), prosomere 2 alar plate (p2A).

Supplementary Figure 5. Developmental growth curves of parent regions

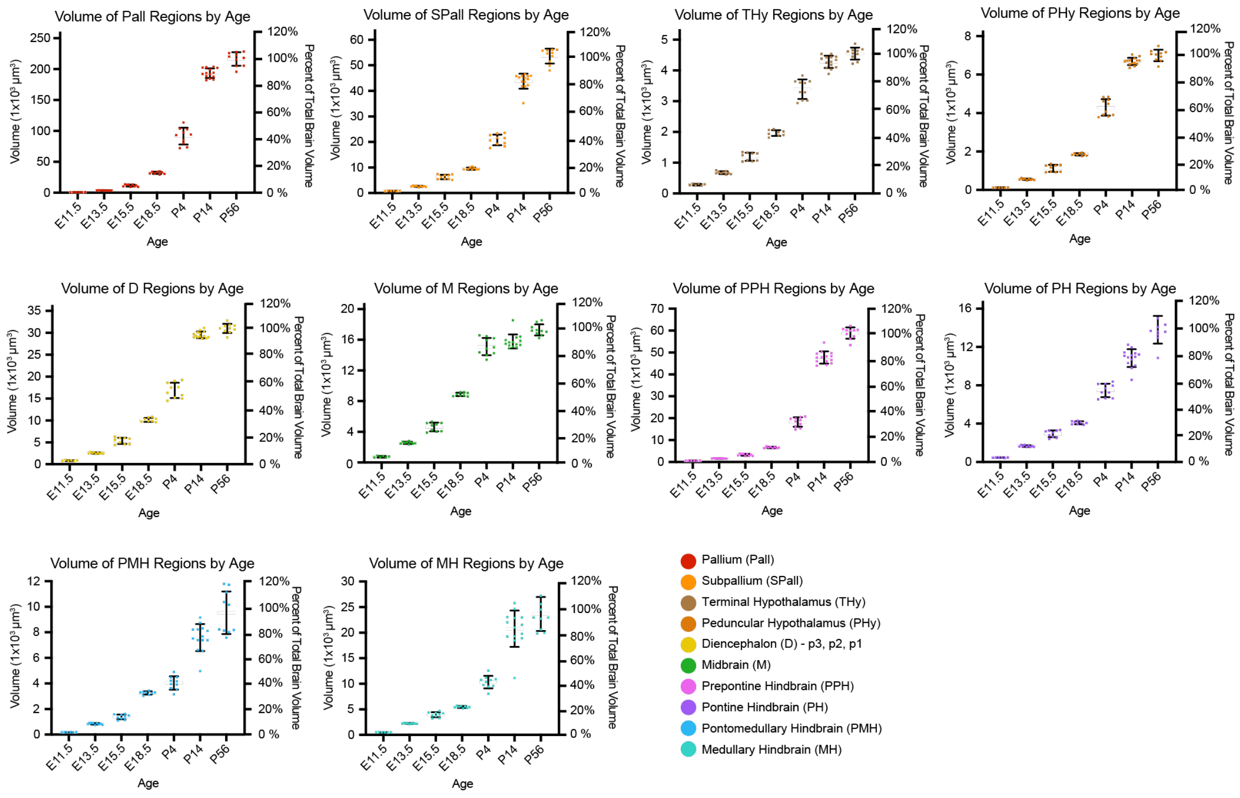

Sample volume and of pallium, subpallium, terminal hypothalamus, peduncular hypothalamus, diencephalon, midbrain, prepontine hindbrain, pontine hindbrain, pontomedullary hindbrain, and medullary hindbrain by age for each template. Regions are colored by DevCCF ontology code. Data is represented as individual sample volume and standard deviation is indicated by error bars. Source data provided in source data file.

Supplementary Figure 6. CCFv3 vs DevCCF Labels

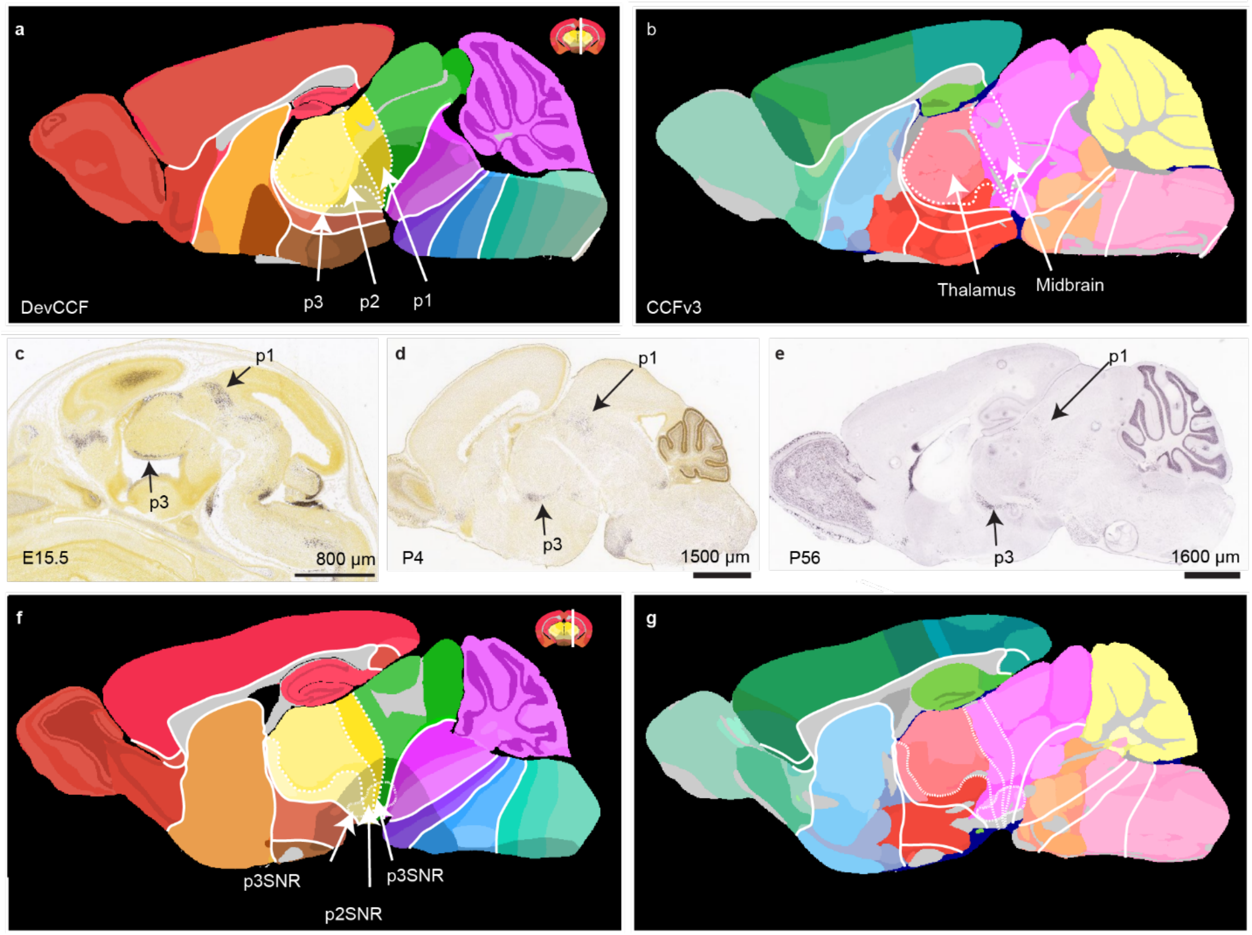

(a) Sagittal section of P56 DevCCF segmentations and (b) corresponding CCFv3 (https://atlas.brain-map.org/) parent regions used for analysis in Fig 5 with annotated DevCCF parent regions overlayed as white lines. Dashed lines represent prosomere (p)1, p2, and p3. (c-e) Diencephalic marker Pax6 expression at E15.5, Allen Mouse Brain Atlas, https://mouse.brain-map.org/experiment/show/100083728 (c), P4, https://mouse.brain-map.org/experiment/show/100032086, (d), and P56, https://mouse.brain-map.org/experiment/show/79677341, (e) denotes DevCCF p1 and p3 as part of the diencephalon through development. (f) Sagittal section of P56 DevCCF segmentations and (g) corresponding CCFv3 (https://atlas.brain-map.org/) parent regions with annotated DevCCF parent regions and substantia nigra pars reticulata (SNR) overlayed as white lines. SNR is divided by diencephalic and midbrain neuromeres in DevCCF (f) compared with a single midbrain region in CCFv3.

Supplementary Figure 7. Anatomical label comparison with molecular atlas

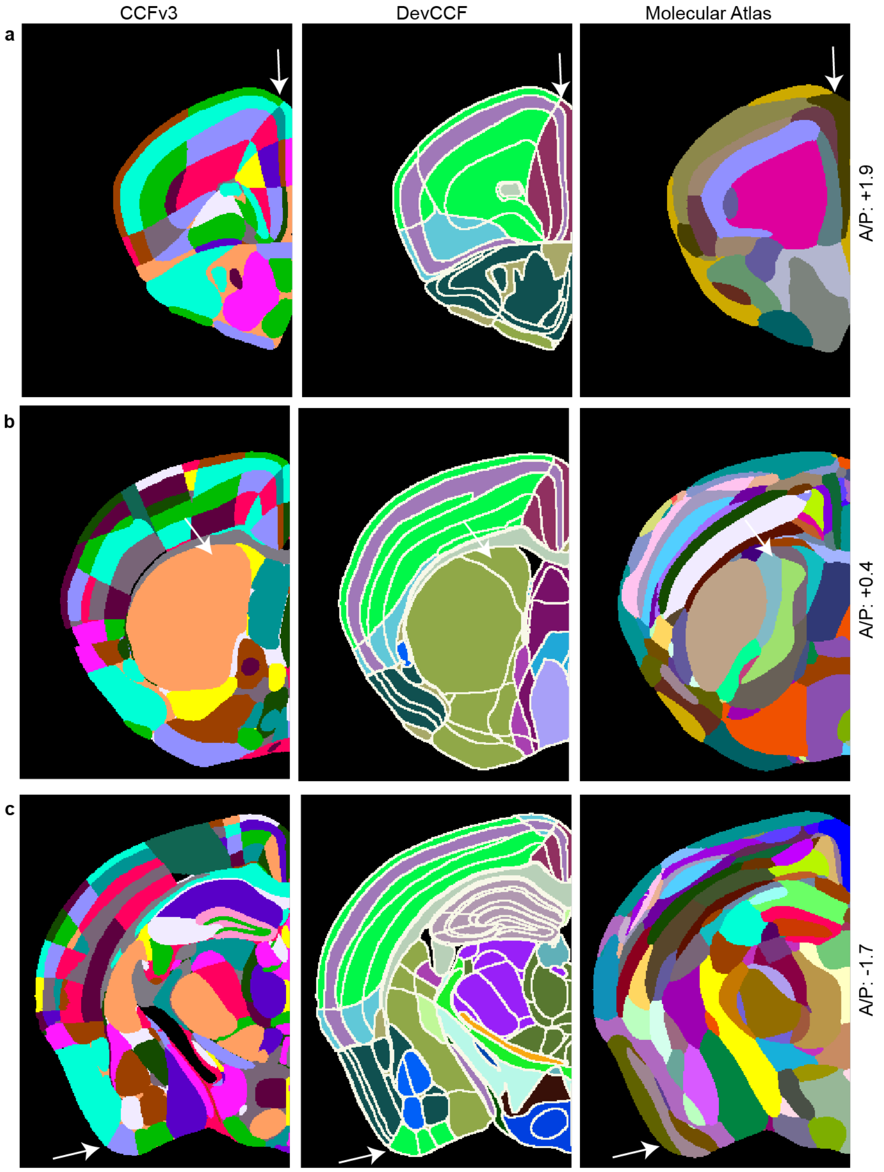

The molecular atlas (https://molecularatlas.org/) based on transcriptome similarity was compared to the CCFv3(https://atlas.brain-map.org/) and DevCCF. (a) The isocortical boundaries showed good agreement such as the boundary between the anterior cingulate and the motor cortex (arrow). (b) While the caudate putamen was treated as a single area in the CCFv3, both DevCCF and the molecular atlas showed subdivisions in the dorsal medial part as indicated by the arrow. (c) While the CCFv3 and DevCCF showed finer segmentation in the piriform and amygdala junction (arrow), the molecular atlas extend the piriform cortex further in the amygdala area. Bregma anterior posterior (A/P) coordinates are indicated in the right side of figures. Atlas labels are semi-randomized to differentiate individual annotations. White lines overlayed on DevCCF represent CCFv3 annotation boundaries. Molecular Atlas panel in the right side column is

derived from the open source atlas data in Cantin Ortiz et al., Molecular atlas of the adult mouse brain (https://github.com/cantin-ortiz/molecular-atlas). Sci. Adv.6,eabb3446(2020).

Supplementary Table 1. DevCCF Version Details

Full Name	Developing Mouse Brain Common Coordinate Framework v1	Developing Mouse Brain Common Coordinate Framework beta3.7	Developing Mouse Brain Common Coordinate Framework beta P56
Short Name	DevCCFv1	DevCCF beta3.7	KimLabDevCCFv001
Version Number	1	0.99	0.98
Version innovation	current manuscript	Preprint https://www.biorxiv.org/content/10.1101/2023.09.14.557789v1	Mendeley Data https://data.mendeley.com/datasets/2svx788ddf/1
Release year	2024	2023	2022
Reference Data	C57BL/6 J population average v1	C57BL/6 J population average v0.99	C57BL/6 J population average v0.98
Coordinate System	DevCCFv1	DevCCFv1	KimLabDevCCFv001
Annotation Set	Whole-brain E11.5, E13.3, E15.5, E18.5, P4, P14, P56	Whole-hemisphere E11.5, E13.3, E15.5, E18.5, P4, P14, P56	Whole-hemisphere P56 only
Terminology (Ontology)	DevCCFv1 ontology	DevCCF beta3.6 ontology	Allen Developing Mouse Brain Ontology
License	Creative Commons Attribution 4.0 International License	Creative Commons Attribution 4.0 International License	Creative Commons Attribution 4.0 International License
Reference	https://doi.org/10.6084/m9.figshare.26377171	https://doi.org/10.1101/2023.09.14.557789	DOI:10.17632/2svx788ddf.1
