# Supplementary material for: Developmental mouse brain common coordinate framework
Source: Nat Commun. 2024 Oct 21;15:9072. doi: 10.1038/s41467-024-53254-w (PMC11494176; doi:10.1038/s41467-024-53254-w)
Supplement: Supplementary file 3 — Description of Additional Supplementary Files [file 41467_2024_53254_MOESM3_ESM.pdf]

Description of Additional Supplementary Files

File name: Supplementary Data 1
Description: Samples used for DevCCF templates. List of subjects used for each template, including age, sex, and imaging modality.

File name: Supplementary Data 2
Description: DevCCF ontology structure. DevCCF ontology structure contains details for each region including DevCCF and ADMBA ID, name, acronym, parent, and color.

File name: Supplementary Data 3
Description: DevCCF vs CCFv3 Voxel Mapping. Number of overlapping voxels of for each P56 DevCCF region and CCFv3 region in P56 DevCCF morphology.

File name: Supplementary Data 4
Description: Key Resource List. List of antibodies, atlases, chemicals, peptides, experimental animal models, and software used to develop the DevCCF.
