# Supplementary material for: Developmental mouse brain common coordinate framework
Source: Nat Commun. 2024 Oct 21;15:9072. doi: 10.1038/s41467-024-53254-w (PMC11494176; doi:10.1038/s41467-024-53254-w)
Supplement: Supplementary file 5 — Reporting Summary [file 41467_2024_53254_MOESM5_ESM.pdf]

nature portfolio

Corresponding author(s): Yongsoo Kim

Last updated by author(s): Aug 5, 2024

Reporting Summary

Nature Portfolio wishes to improve the reproducibility of the work that we publish. This form provides structure for consistency and transparency in reporting. For further information on Nature Portfolio policies, see our Editorial Policies and the Editorial Policy Checklist.

Statistics

For all statistical analyses, confirm that the following items are present in the figure legend, table legend, main text, or Methods section.

n/a	Confirmed	
☐	☒	The exact sample size (*n*) for each experimental group/condition, given as a discrete number and unit of measurement
☐	☒	A statement on whether measurements were taken from distinct samples or whether the same sample was measured repeatedly
☒	☐	The statistical test(s) used AND whether they are one- or two-sided<br>*Only common tests should be described solely by name; describe more complex techniques in the Methods section.*
☒	☐	A description of all covariates tested
☒	☐	A description of any assumptions or corrections, such as tests of normality and adjustment for multiple comparisons
☐	☒	A full description of the statistical parameters including central tendency (e.g. means) or other basic estimates (e.g. regression coefficient) AND variation (e.g. standard deviation) or associated estimates of uncertainty (e.g. confidence intervals)
☒	☐	For null hypothesis testing, the test statistic (e.g. *F*, *t*, *r*) with confidence intervals, effect sizes, degrees of freedom and *P* value noted<br>*Give P values as exact values whenever suitable.*
☒	☐	For Bayesian analysis, information on the choice of priors and Markov chain Monte Carlo settings
☒	☐	For hierarchical and complex designs, identification of the appropriate level for tests and full reporting of outcomes
☒	☐	Estimates of effect sizes (e.g. Cohen's *d*, Pearson's *r*), indicating how they were calculated

Our web collection on statistics for biologists contains articles on many of the points above.

Software and code

Policy information about availability of computer code

Data collection

SmartSPIM (LifeCanvas Technologies, Cambridge, MA, USA) was used to collect LSFM Data
7 Tesla MRI system (Bruker Biospin, Billerica, MA, USA) was used to collected undistorted brain imaging data.

Data analysis

MATLAB R2022a LSFM image tile stitching, MRI k-space reconstruction
Applied Normalization Toolbox (ANTs) v2.3.5: Template generation, image registration
ITK-SNAP v4: Image visualization
Python v3.8.16: ISH registration to DevCCF (with ANTsPy, NumPy, and Pandas)
Python v3.11: Plotting CCFv3 vs DevCCF comparisons (with Pandas, Seaborn, Plotly)
ANTsPy v0.3.8: Ad-hoc image processing (applying masks, combining annotat
ions, etc.)
Dragonfly 2022.2: Molecular Atlas conversion to voxels
NumPy v1.21.1: Supplement to ANTsPy
Seaborn v0.12: Plotting in Python
Plotly v5.10 Plotting in python
Avizo v2021.2: Atlas segmentation
ilastik v1.3.3post3: GAD2 positive pixel classification.
Neuroglancer v3.5: Web visualization development
Allen Brain Atlas API: ISH data download
Pandas v2.2.0: Supplement to ANTsPy
Prism v: Plot volumetric growth curves

DTI Studio v1.8: Diffusion tensor image construction

Custom Code:
DevCCF Code : Template generation, multimodal registration, LSFM stitching, 2D ISH to 3D DevCCF registration
https://doi.org/10.5281/zenodo.12853683

DevCCF CCFv3 mapping code: Create DevCCF vs CCFv3 Sankey diagram
https://doi.org/10.5281/zenodo.12854081

DevCCFv1 Dataset Reference (FigShare Download)
https://doi.org/10.6084/m9.figshare.26377171

For manuscripts utilizing custom algorithms or software that are central to the research but not yet described in published literature, software must be made available to editors and reviewers. We strongly encourage code deposition in a community repository (e.g. GitHub). See the Nature Portfolio guidelines for submitting code & software for further information.

Data

Policy information about availability of data

All manuscripts must include a data availability statement. This statement should provide the following information, where applicable:

- Accession codes, unique identifiers, or web links for publicly available datasets
- A description of any restrictions on data availability
- For clinical datasets or third party data, please ensure that the statement adheres to our policy

The DevCCF [https://doi.org/10.6084/m9.figshare.26377171]105 and unwarped LSFM templates [https://doi.org/10.6084/m9.figshare.26492122]106 generated in this manuscript have been deposited in FigShare. The DevCCF has been assigned RRID:SCR_025544. The DevCCF interactive viewer is available via the DevCCF hub [https://kimlab.io/brain-map/DevCCF/]. Individual sample MRI and LSFM datasets used to generate templates have been deposited in the Brain Imaging Library, accessible by searching for grant number RF1-MH124605 (https://api.brainimagelibrary.org/web/). CCFv3 and ADMBA datasets are available via the Allen Brain Atlas API [https://mouse.brain-map.org/static/api]. The Molecular Atlas is available at https://molecularatlas.org/. Source data for Fig. 4g, Fig. 5i, Fig. 6c, Fig. 6e, and Supplementary Fig. 5 are provided with the source data file. Supplementary Data files are provided with the supplementary data file. The Developmental Common Coordinate Framework (DevCCF) is an openly accessible resource via a Creative Commons Attribution 4.0 International License. Template, reference, annotation, and ontology version information are summarized in Supplementary Table 1. Additional information is available by contacting the corresponding author, Yongsoo Kim (yuk17@psu.edu).

Research involving human participants, their data, or biological material

Policy information about studies with human participants or human data. See also policy information about sex, gender (identity/presentation), and sexual orientation and race, ethnicity and racism.

Reporting on sex and gender	This research does not involve human participants, their data, or biological material.
Reporting on race, ethnicity, or other socially relevant groupings	This research does not involve human participants, their data, or biological material.
Population characteristics	This research does not involve human participants, their data, or biological material.
Recruitment	This research does not involve human participants, their data, or biological material.
Ethics oversight	This research does not involve human participants, their data, or biological material.

Note that full information on the approval of the study protocol must also be provided in the manuscript.

Field-specific reporting

Please select the one below that is the best fit for your research. If you are not sure, read the appropriate sections before making your selection.

☒ Life sciences ☐ Behavioural & social sciences ☐ Ecological, evolutionary & environmental sciences

For a reference copy of the document with all sections, see nature.com/documents/nr-reporting-summary-flat.pdf

Life sciences study design

All studies must disclose on these points even when the disclosure is negative.

Sample size	We used 6 - 14 samples per age to create populated averaged atlas templates. We use similar number of both male and females in our samples.
Data exclusions	MRI and LSFM 3D imaging data with poor imaging quality were excluded from the results. Data was assessed for anatomical abnormalities,

Data exclusions	imaging artifacts, staining or fluorescence issues (where appropriate), or brain damage from sample preparation and acquisition. Data quality for each dataset was scored on a scale from 1 to 5, where 1 is lowest quality and 5 is the highest quality. Data quality of 3 or lower was excluded from template generation. Data quality 2 or lower was excluded from cell type analysis.<br><br>1 (Poorest Quality): Severe abnormalities, artifacts, staining/fluorescence issues, or damage to whole brain<br>2: Severe abnormalities, artifacts, staining/fluorescence issues, or damage with some regions remaining intact<br>3: Moderate abnormalities, artifacts, staining/fluorescence issues, or damage with some regions remaining intact<br>4: Minor abnormalities, artifacts, staining/fluorescence issues, or damage to few regions<br>5 (Highest Quality): No abnormalities, artifacts, staining/fluorescence issues, or damage to brain sample
Replication	Detailed methods and data are provided in the manuscript to enable study replication. Resources used are detailed in Supplementary Data 4.
Randomization	Samples were grouped by age. Randomization was not relevant to this study as there are no experimental groups.
Blinding	Blinding was not relevant to this study as there are no experimental groups.

Reporting for specific materials, systems and methods

We require information from authors about some types of materials, experimental systems and methods used in many studies. Here, indicate whether each material, system or method listed is relevant to your study. If you are not sure if a list item applies to your research, read the appropriate section before selecting a response.

Materials & experimental systems

n/a	Involved in the study	
☐	☒	Antibodies
☒	☐	Eukaryotic cell lines
☒	☐	Palaeontology and archaeology
☐	☒	Animals and other organisms
☒	☐	Clinical data
☒	☐	Dual use research of concern
☒	☐	Plants

Methods

n/a	Involved in the study	
☒	☐	ChIP-seq
☒	☐	Flow cytometry
☐	☒	MRI-based neuroimaging

Antibodies

Antibodies used	Mouse Monoclonal Antibody to Neurofilament NF-H<br>Clone Name: Monocolonal Antibody<br>Source: EnCor Biotechnology Inc.<br>Amount/dilution: 10 µl per hemisphere / 1:100 dilution<br>Cat. no. MCA-9B12<br>Lot Number: 012022<br>RRID: AB_2572358<br><br>Alexa Fluor® 647 AffiniPure™ Fab Fragment Donkey Anti-Mouse IgG (H+L)<br>Clone Name: Polyclonal Antibody<br>Source: Jackson Immuno Research<br>Amount/Dilution: 2.7 µl per hemisphere / 1:500 dilution<br>Cat. no.: 715-607-003<br>Lot Number: 153490<br>RRID: AB_2340867
Validation	We performed no primary antibody control experiment and confirmed lack of non-specific staining. The vendor also provides the specificity information of the antibody; https://encorbio.com/product/mca-9b12/

Animals and other research organisms

Policy information about studies involving animals; ARRIVE guidelines recommended for reporting animal research, and Sex and Gender in Research

Laboratory animals	C57bl/6J mice (The Jackson Laboratory, RRID: IMSR_JAX:000664, Ages E11.5, E13.5, and E15.5, E18.5, P4, P14, and P56)<br>Cre-dependent tdTomato fluorescent reporter mice (9The Jackson Laboratory, RRID: IMSR_JAX:007908, Ages E11.5, E13.5, and E15.5)<br>Gad2-IRES-Cre mice (The Jackson Laboratory, RRID: IMSR_JAX:028867, Ages E11.5, E13.5, and E15.5)<br><br>All mice were maintained under a 12-hour light/12-hour dark cycle at 22–25°C with access to food and water ad libitum.<br>Adult breeder males were singly housed for 1 week prior to pairing.

Wild animals	This study did not involve wild animals.
Reporting on sex	DevCCF templates are morphological averages of male and female sex animals. We used tail samples with PCR for genotyping including Rbm31-based sex genotyping for mice age postnatal day (P)4 and younger. P14 and P56 mice sex was determined based on the presence of internal genitals (females) or external genitals (males). Of 135 samples, we identified 62 from male specimens, 57 from female specimens, and 16 specimens for which sex was not obtained. Breakdown by age and modality is found in Extended Data Table 1.
Field-collected samples	This study did not involve field-collected samples
Ethics oversight	All experiments and techniques involving live animals have been approved and conform to the regulatory standards set by the Institutional Animal Care and Use Committee (IACUC) at the Pennsylvania State University College of Medicine.

Note that full information on the approval of the study protocol must also be provided in the manuscript.

Magnetic resonance imaging

Experimental design

Design type	Ex vivo imaging to obtain structural information
Design specifications	Single acquisition from fixed brains
Behavioral performance measures	N/A

Acquisition

Imaging type(s)	structural
Field strength	7 Tesla
Sequence & imaging parameters	echo time (TE)/repetition time (TR) = 30/400 ms, two signal averages, diffusion gradient duration/separation = 4/12 ms, 60 diffusion directions with a b-value of 1.0 ms/μm2 for E11.5, 2.0 ms/μm2 for E13.5-E17.5, and 5.0 ms/μm2 for P4, P14, and P56 brains.
Area of acquisition	Whole Brain Scan was used

Diffusion MRI ☒ Used ☐ Not used

Parameters: echo time (TE)/repetition time (TR) = 30/400 ms, two signal averages, diffusion gradient duration/separation = 4/12 ms, 60 diffusion directions with a b-value of 1.0 ms/μm2 for E11.5, 2.0 ms/μm2 for E13.5-E17.5, and 5.0 ms/μm2 for P4, P14, and P56 brains. The increase in b-values with age was necessary as the diffusivity of brain tissues decreases with development

Preprocessing

Preprocessing software	The 3D MRI data were reconstructed from k-space to images and zero-padded to twice the raw image resolution in each dimension in MATLAB (Mathworks, Natick, MA, USA). Magnetization transfer ratio map was computed using MTR = 1-MT/M0. Diffusion tensor images70 were constructed using the log-linear fitting method in DTI Studio (http://www.mristudio.org), and the tensor-based scalar metrics were generated, including the mean diffusivity (MD) and fractional anisotropy (FA).
Normalization	Individual 3D imaging data were normalized to a morphological group average using linear and non-linear registration methods. Images were stored in the compressed Nifti (.nii.gz) format.
Normalization template	Applied Normalization Tools (ANTs) Version 2.3.5 was used to generate unique templates and perform image registration at each age. Futher proceedures were performed in DevCCF group standardized space
Noise and artifact removal	*Describe your procedure(s) for artifact and structured noise removal, specifying motion parameters, tissue signals and physiological signals (heart rate, respiration).*
Volume censoring	*Define your software and/or method and criteria for volume censoring, and state the extent of such censoring.*

Statistical modeling & inference

Model type and settings	Statistical modeling was not performed on MRI data
Effect(s) tested	Statistical modeling was not performed on MRI data

Specify type of analysis: ☒ Whole brain ☐ ROI-based ☐ Both

Statistic type for inference (See Eklund et al. 2016)	Statistical modeling was not performed on MRI data
Correction	Statistical modeling was not performed on MRI data

Models & analysis

n/a	Involved in the study
☒	☐ Functional and/or effective connectivity
☒	☐ Graph analysis
☒	☐ Multivariate modeling or predictive analysis
